# Development of a Novel σ_1_ Receptor Biosensor Based on Its Heterodimerization with Binding Immunoglobulin Protein in Living Cells

**DOI:** 10.1021/acschemneuro.3c00206

**Published:** 2023-05-16

**Authors:** Xavier Morató, Víctor Fernández-Dueñas, Pilar Pérez-Villamor, Marta Valle-León, José Miguel Vela, Manuel Merlos, Javier Burgueño, Francisco Ciruela

**Affiliations:** † Pharmacology Unit, Department of Pathology and Experimental Therapeutics, Faculty of Medicine and Health Sciences, Institute of Neurosciences, University of Barcelona, 08907 L’Hospitalet de Llobregat, Spain; ‡ Neuropharmacology and Pain Group, Neuroscience Program, Bellvitge Biomedical Research Institute, IDIBELL, 08908 L’Hospitalet de Llobregat, Spain; § Welab Barcelona, Parc Científic Barcelona, 08028 Barcelona, Spain

**Keywords:** σ_1_ receptor, binding immunoglobulin protein, PRE-084, oligomerization, haloperidol, biosensor

## Abstract

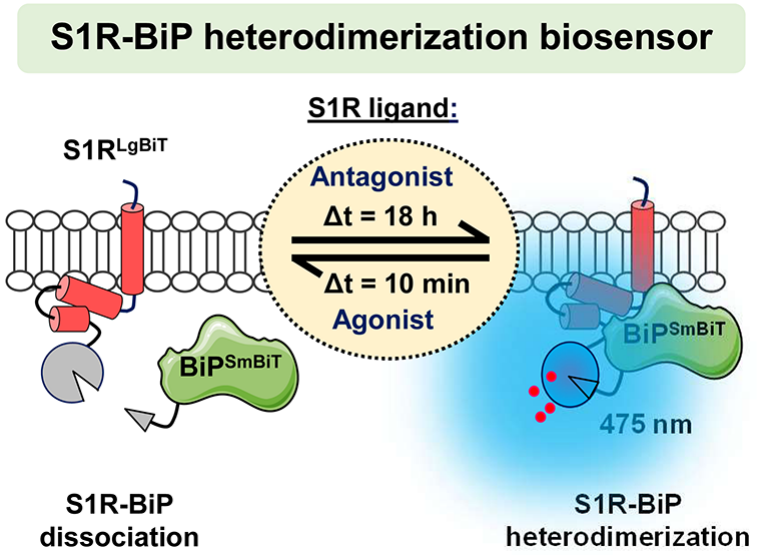

The σ_1_ receptor (S1R) is a ligand-regulated non-opioid intracellular receptor involved in several pathological conditions. The development of S1R-based drugs as therapeutic agents is a challenge due to the lack of simple functional assays to identify and classify S1R ligands. We have developed a novel nanoluciferase binary technology (NanoBiT) assay based on the ability of S1R to heteromerize with the binding immunoglobulin protein (BiP) in living cells. The S1R-BiP heterodimerization biosensor allows for rapid and accurate identification of S1R ligands by monitoring the dynamics of association–dissociation of S1R and BiP. Acute treatment of cells with the S1R agonist PRE-084 produced rapid and transient dissociation of the S1R-BiP heterodimer, which was blocked by haloperidol. The effect of PRE-084 was enhanced by calcium depletion, leading to a higher reduction in heterodimerization even in the presence of haloperidol. Prolonged incubation of cells with S1R antagonists (haloperidol, NE-100, BD-1047, and PD-144418) increased the formation of S1R-BiP heteromers, while agonists (PRE-084, 4-IBP, and pentazocine) did not alter heterodimerization under the same experimental conditions. The newly developed S1R-BiP biosensor is a simple and effective tool for exploring S1R pharmacology in an easy cellular setting. This biosensor is suitable for high-throughput applications and a valuable resource in the researcher’s toolkit.

## Introduction

The σ_1_ receptor (S1R), initially recognized as a new subtype of opioid receptor,^1^ was cloned in 1996^2^ and subsequently classified as a non-opioid and even non-G-protein-coupled receptor (GPCR) (for review, see ref (3)). In 2013, the International Union of General and Clinical Pharmacology cataloged S1R as a ligand-regulated non-opioid intracellular receptor.^4^ Since then, evidence has been provided supporting participation of S1R in various pathological conditions, such as pain, cardiovascular disease, cancer, drug addiction, or neurodegenerative disorders.^5−7^ Consequently, although no apparent endogenous ligand has been unambiguously identified, efforts have been made to develop S1R compounds as therapeutic agents.

The S1R does not have a defined signaling pathway; instead, the dominant accepted model is that the S1R modulates other cellular signaling pathways by acting as a ligand-operated chaperone.^8^ In fact, many protein–protein interactions involving S1R and other partners, such as voltage- or ligand-gated ion channels, GPCRs, transporters, or enzymes, have been identified, supporting its chaperone-like activity.^3^ Importantly, the ability of S1R to homo- and heteromerize can be regulated by ligands.^9^ Therefore, while S1R antagonists favor the formation of higher-order receptor oligomers, agonists promote the opposite, namely, the generation of lower-molecular-weight forms, such as homodimeric or monomeric receptors. In fact, the regulation of S1R oligomerization by ligands constitute the basis for considering this receptor as a ligand-operated chaperone. Specifically, at the interface between the endoplasmic reticulum (ER) and the mitochondrion (mitochondria-associated ER membrane, MAM), S1R interacts with the binding immunoglobulin protein (BiP), a resident chaperone of ER.^3,10−12^ Specifically, S1R agonists or a reduction in ER calcium levels prompts the dissociation of S1R and BiP, which disclose the sole intrinsic chaperone activity of S1R and BiP with their respective client proteins.^8^ Consistent with this, S1R is a calcium-sensitive chaperone located in the ER, specifically in the MAM, where it exerts an important role in stabilizing this interorganelle region, calcium homeostasis, mitochondrial bioenergetics, and ER stress response.^10,13^ In addition, S1R can eventually translocate to the plasma membrane where it interacts with ion channels and GPCR. Finally, S1R can also be found in the nuclear envelope, where it regulates transcription.^10,11^

The S1R has a very broad pharmacological profile. Thus, this receptor binds to ligands of different chemical structures and pharmacological actions, including antipsychotics (haloperidol), analgesics (pentazocine), or even narcotic drugs (cocaine).^14−16^ The pharmacological classification of these ligands has been largely based on *in vivo* animal models, with S1R antagonists defined as ligands that recapitulate the phenotype of *SIGMAR1* gene knockdown and that can attenuate the effects of S1R stimulation (i.e., hyperlocomotion). On the contrary, S1R agonists are defined as ligands that recreate a phenotype similar to receptor overexpression.^17^ This classification is useful in terms of therapeutics but does not allow for a simple and effective way to explore S1R pharmacology. Consequently, there is an urgent need for effortless and high-throughput assays that allow unambiguous stratification of S1R ligands according to their expected intrinsic activity (i.e., agonist and antagonist) *in vivo*. Based on this premise, our objective consisted of developing an *in vitro* assay, which would be implemented in the discovery of S1R drugs. To this end, here we engineered a S1R heteromerization biosensor for *in cellulo* pharmacological research using highly sensitive nanoluciferase (NLuc) binary technology (NanoBiT). Then, we evaluated the impact of S1R ligands on the heterodimerization of S1R and BiP, to classify them as S1R agonists or antagonists.

## Results

### Engineering a NanoBiT-Based S1R-BiP Heterodimerization Biosensor

**Figure 1:**
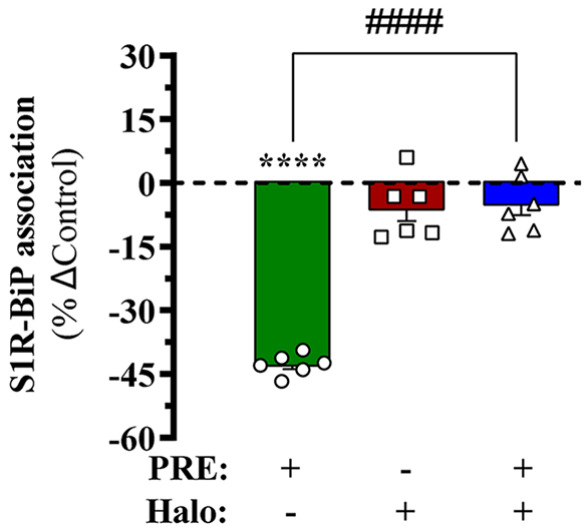
Effect of S1R ligands on receptor-BiP association in CHO cells. CHO cells were incubated with PRE-084 (10 μM, PRE) in the absence or presence of haloperidol (10 μM, Halo) for 30 min. The endogenous S1R and BiP were coimmunoprecipitated using a S1R antibody, and BiP levels were measured by ELISA. The results are represented as percentage of difference against CoIP-ELISA values determined in vehicle treated cells (% ΔBasal) and expressed as the mean ± SEM (*n* = 6): ****p < 0.0001 one-way ANOVA with Dunnett’s post hoc test when compared to vehicle-treated cells (dashed line) and ^####^*p* < 0.0001 with Tukey’s post hoc test.

The formation of S1R and BiP complexes and the regulation of their association by ligands were previously demonstrated using a coimmunoprecipitation (CoIP) coupled enzyme-linked immunosorbent assay (ELISA).^18^ Therefore, the S1R agonist PRE-084 promoted the dissociation of the S1R-BiP complex, which was blocked by NE-100, a S1R antagonist. Here, using the same S1R-BiP association assay from Amylgen,^18^ we were able to reproduce once again the ability of PRE-084 to promote the dissociation of the S1R-BiP complex. Furthermore, haloperidol, a S1R antagonist, blocked PRE-084-induced S1R-BiP dissociation (Figure 1), as previously reported.^8^ However, we realized that the S1R-BiP association CoIP-ELISA presents challenges in terms of scalability in a high-throughput format. Moreover, the commercial discontinuation of this assay led us to develop a novel and reliable procedure to accurately classify S1R ligands.

**Figure 2:**
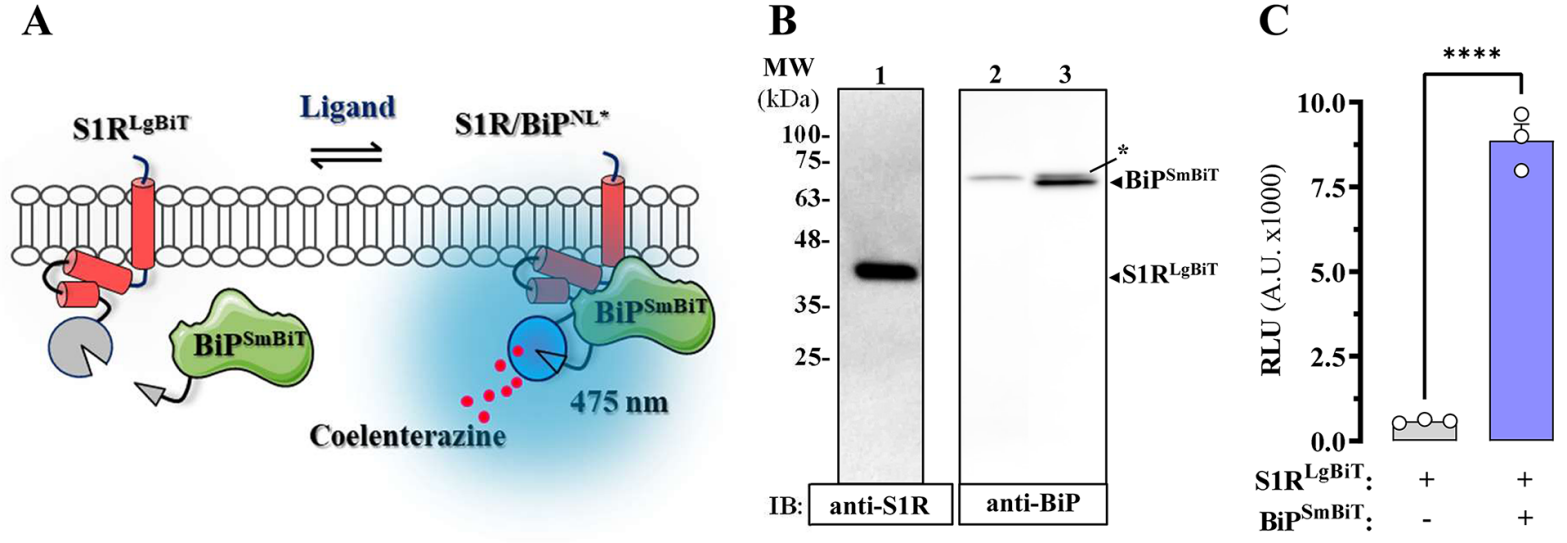
S1R-BiP heterodimer biosensor. (A) Schematic representation of the specific NanoBiT-based protein–protein interaction assay designed to monitor S1R-BiP heterodimerization dynamics. S1R tagged with the LgBiT and the BiP with the SmBiT fragments of the NanoLuciferase (NL) enzyme (i.e., S1RLgBiT and BiPSmBiT, respectively) were designed. Only when SmBiT and LgBiT are in close proximity (i.e., S1R-BiP heterodimerization) did these fragments render the active NL, which upon incubation with coelenterazine will generate light at 475 nm. Figure was designed using Servier Medical Art image templates (https://smart.servier.com/image-set-download/). (B) Immunoblot showing S1R and BiP expression in living cells. Representative immunoblot showing the expression of S1R in membrane extracts (10 μg) from HEK- 293S1R-KO cells permanently transfected with S1RLgBiT (lanes 1 and 2) and S1RLgBiT plus BiPSmBiT (lane 3) constructs. Membrane extracts were analyzed by SDS–PAGE and immunoblotted using rabbit anti-S1R or mouse anti-BiP (see Methods). The asterisk denotes the endogenous BiP protein. (C) S1R-BiP heterodimermediated NL complementation. HEK-293T cells permanently expressing S1RLgBiT in the absence or presence of BiPSmBiT were incubated with coelenterazine 400a (1 μM), and the luminescence was recorded. The results of three independent experiments carried out in triplicate were expressed as the mean ± SEM (*n* = 3) of the relative luminescence signal (RLU): ****p < 0.0001 Student’s *t* test.

We engineered an intermolecular biosensor using NanoBiT technology^19^ to monitor dynamic changes in S1R and BiP heterodimerization. To this end, the large 18 kDa split fragment of NLuc (LgBit) was fused to S1R, while the small 1.3 kDa split fragment (SmBit) was fused to BiP (Figure 2A). Subsequently, we generated a HEK-293T cell line permanently expressing S1R^LgBiT^ and BiP^SmBiT^ (i.e., S1R-BiP heterodimerization biosensor) (Figure 2). Importantly, the HEK-293T cell line used to generate the biosensor lacked S1R (see Methods), thus avoiding any potential interference of the endogenous receptor. The permanent expression of S1R^LgBiT^ and BiP^SmBiT^ at the protein level was monitored by immunoblot using specific antibodies (Figure 2B). The ability of NL to reconstitute after S1R-BiP heterodimerization in HEK-293T cells was evaluated by recording NL-mediated luminescence (Figure 2C). Collectively, these results validated our NanoBiT-based approach to further monitor the heterodimerization of S1R with BiP in living cells.

### Acute S1R Ligand-Mediated Modulation of the S1R-BiP Heterodimerization Biosensor

**Figure 3:**
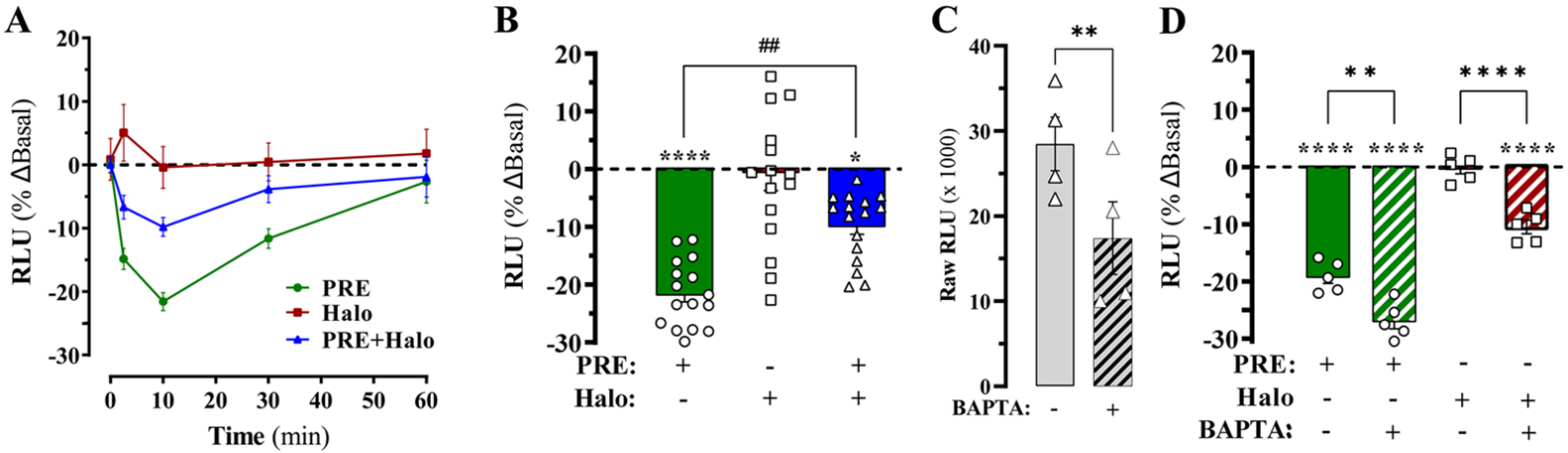
Acute ligand-mediated modulation of S1R-BiP heterodimerization. (A) Time-course ligand-mediated modulation of S1R-BiP heterodimerization. The S1RLgBIT/BiPSmBIT HEK-293 stable cell line was first incubated with coelenterazine to assess basal luminescence and thereafter challenged with vehicle (dashed line) or the indicated S1R ligands (10 μM) during 60 min. Luminiscence (RLU) was recorded at different time points to assess S1R-BiP heterodimerization. The results are represented as a percentage of difference with vehicle basal luminescence (% ΔBasal) over time and expressed as the mean ± SEM of four independent experiments performed in quadruplicate. (B) Quantification of the luminiscence (RLU) peak observed at 10 min shown in panel A. *p < 0.05; ****p < 0.0001 one-way ANOVA with Dunnett’s post hoc test when compared to vehicle-treated cells (dashed line) and ^##^*p* < 0.01 with Tukey’s post hoc test. (C) Effect of calcium depletion on S1R-BiP heterodimerization. The S1RLgBIT/BiPSmBIT HEK-293 stable cell line was preincubated in the absence or presence of BAPTA-AM (10 μM) for 30 min before adding coelenterazine to assess basal luminescence (Raw RLU). Data are expressed as the mean ± SEM of four independent experiments performed in quadruplicate. **p < 0.01, Student’s *t* test. (D) Effect of calcium depletion on S1R ligand-mediated modulation of S1R-BiP heterodimerization. The S1RLgBIT/BiPSmBIT HEK-293 stable cell line was preincubated in the absence or presence of BAPTA-AM (10 μM) before the indicated S1R ligands (10 μM) were added during 60 min as described in panel A. The luminescence 10 min peak was quantified as in panel B. Data are expressed as the mean ± SEM of five independent experiments performed in quadruplicate: **p < 0.01, ****p < 0.0001, two-way ANOVA with Sidak’s post hoc test. PRE-084 (PRE), haloperidol (Halo).

Subsequently, the impact of S1R ligands on S1R-BiP heterodimerization was evaluated by acutely treating HEK-293T cells expressing the S1R-BiP biosensor with agonists or antagonists. To this end, time-course experiments were performed monitoring NL luminescence in S1R^LgBiT^-BiP^SmBiT^ HEK-293T cells challenged with PRE-084 and haloperidol (Figure 3A). Interestingly, PRE-084 produced a time-dependent reduction in S1R-BiP heterodimerization with a peak at 10 min followed by a recovery until normality, which was achieved at 60 min (Figure 3A). On the contrary, the treatment with haloperidol, under the same experimental conditions, did not have an effect on heterodimerization (Figure 3A). It is noteworthy that after 10 min of incubation, PRE-084 led to a significant reduction (21.6 ± 1.4%, *p* < 0.0001), while haloperidol did not affect heterodimerization of S1R and BiP (*p* = 0.9984) (Figure 3B). Importantly, incubation of S1R^LgBiT^-BiP^SmBiT^ HEK-293T cells with haloperidol partially, but significantly (*p* = 0.0018), blocked the PRE-084 induced reduction in heterodimerization (Figure 3B). On the other hand, treatment of S1R^LgBiT^-BiP^SmBiT^ HEK-293T cells with BAPTA-AM, which depletes both cytosolic and ER Ca^2+^, significantly reduced heterodimerization (Figure 3C,D). A two-way ANOVA (S1R ligand × BAPTA-AM) revealed a significant main effect of S1R ligand treatment (*F*_(1,16)_ = 210.9, *p* < 0.0001), BAPTA-AM treatment (*F*_(1,16)_ = 54.91, *p* < 0.0001) but not the interaction between both factors (*F*_(1,16)_ = 1.104, *p* = 0.3091). These results indicated that both intracellular calcium depletion and treatment with a putative S1R agonist promoted the dissociation of the S1R-BiP heterodimer. Furthermore, while the S1R antagonist did not alter the overall heterodimer content, it was able to block the dissociation of the S1R-BiP heterodimer induced by the agonist.

### Prolonged Ligand-Mediated Modulation of the NanoBiT-Based S1R-BiP Heterodimerization Biosensor

**Figure 4:**
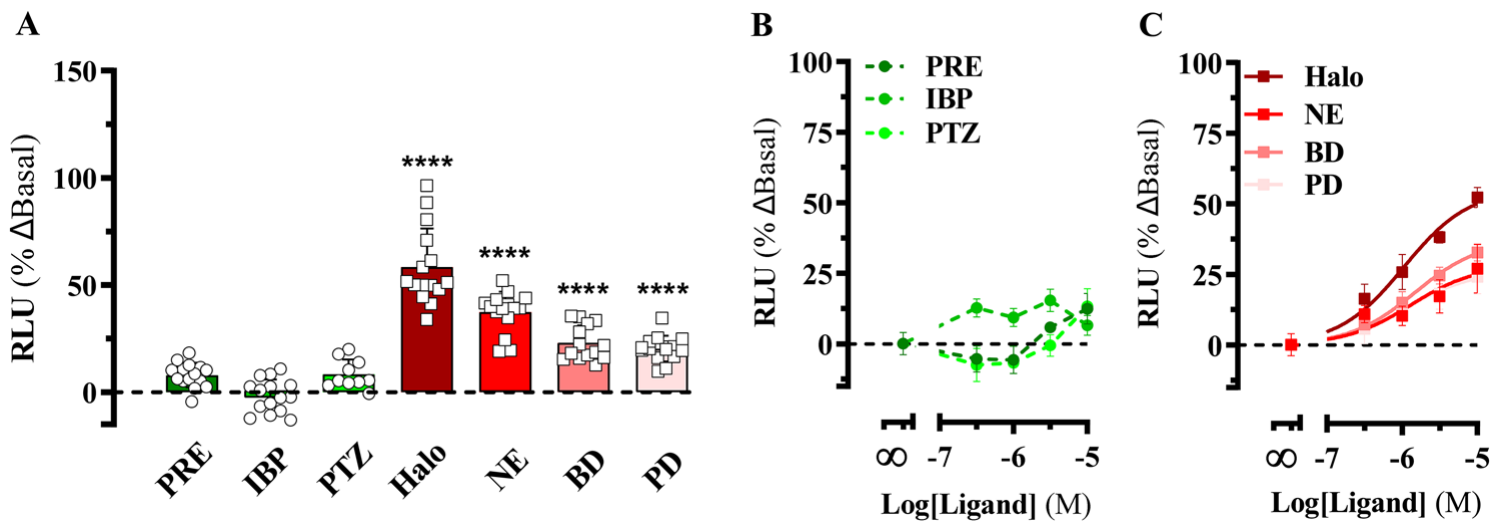
Prolonged S1R ligand-mediated modulation of S1R-BiP heterodimerization. (A) The stable S1RLgBIT/BiPSmBIT HEK-293 cell line was incubated with vehicle or the indicated S1R ligand (10 μM) during 16 h before the S1R-BiP heterodimerization was determined after incubation with 1 μM coelenterazine 400a for 15 min, and the end point luminescence recordings were assessed on a CLARIOstar microplate reader. The results are expressed as the mean ± SEM of three independent experiments performed in quintuplicate: ****p < 0.0001 one-way ANOVA with Dunnett’s post hoc test compared to vehicle-treated cells. Concentration–response experiments treating S1RLgBIT/BiPSmBIT HEK-293 cells with increasing concentrations of a series of putative agonists (B) and antagonist (C) for the S1R were performed as described in panel A. The results are represented as percentage of difference against vehicle basal luminescence (% ΔBasal) and expressed as the mean ± SEM of three independent experiments performed in quintuplicate: ****P < 0.0001 one-way ANOVA with Dunnett’s post hoc test when compared to vehicle-treated cells (dashed line). Haloperidol (Halo), PRE-084 (PRE), NE-100 (NE), pentazocine (PTZ), BD-1047 (BD), PD-144418 (PD), and 4-IBP.

Once we validated the S1R-BiP heterodimer biosensor to study acute modulation of the S1R and BiP interaction in living cells, we aimed to investigate unexplored experimental conditions more suitable for high-throughput processes. To this end, we challenged the S1R-BiP heterodimer biosensor with a series of putative agonists (i.e., PRE-084, 4-IBP, and pentazocine) and antagonists (i.e., haloperidol, NE-100, BD-1047, and PD-144418) for a 16 h (overnight) incubation period, after which we performed a single end point luminescence determination. Interestingly, challenging cells with 10 μM S1R agonists did not affect S1R-BiP heterodimerization, while incubation with S1R antagonists significantly promoted the interaction of S1R and BiP (Figure 4A). Subsequently, the concentration–response curves were constructed by incubating S1R^LgBiT^ and BiP^SmBiT^ expressing HEK-293T cells with increasing concentrations of S1R ligands. Haloperidol promoted the highest increase in S1R-BiP heteromerization (*E*_max_ = 55 ± 3% and pEC_50_ = 6 ± 0.1) when compared to NE-100 (*E*_max_ = 28 ± 4% and pEC_50_ = 5.9 ± 0.2), BD-1047 (*E*_max_ = 37 ± 2% and pEC_50_ = 5.8 ± 0.1), or PD-144418 (*E*_max_ = 26 ± 3% and pEC_50_ = 5.9 ± 0.2) (Figure 3 B). In contrast, the S1R agonists (i.e., PRE-084, 4-IBP, and pentazocine) did not show a concentration-dependent effect on the formation of the S1R-BiP heteromers (Figure 3A). In general, these results demonstrated that S1R agonists were ineffective in promoting S1R-BiP heterodimerization, while antagonists potentiated S1R-BiP heteromer formation. These results suggested a unique mechanism of action for S1R antagonists in receptor heterodimerization after 16 h of incubation.

## Discussion

The search for effective S1R drugs has been hampered by the lack of unbiased functional assays capable of accurately identifying and classifying S1R ligands. Consequently, candidate drugs are frequently selected based on their performance in preclinical animal models, which are time-consuming and expensive and often do not provide a clear pharmacological profile. As a result, the development of effective S1R drugs is a challenging task, highlighting the need for new pharmacological assays to streamline the drug discovery process. In addition, reducing the dependence on *in vivo* models may result in a more ethical and sustainable approach in animal research. Here, we present a novel *in cellulo* assay based on S1R-BiP heterodimerization, which allows differentiating S1R ligands with putative agonistic or antagonistic properties. Therefore, we engineered a biosensor based on the ligand-operated capacity of S1R to interact with BiP.^3,11^ Acute treatment of cells expressing the biosensor with a putative S1R agonist produced rapid and transient dissociation of the S1R-BiP heterodimer, which was blocked by an antagonist. However, prolonged incubation with S1R antagonists potentiated the formation of S1R-BiP heteromers, while agonists under the same experimental conditions did not alter the heterodimer content. Interestingly, this last experimental setting makes the new S1R-BiP heteromerization assay more suitable for high-throughput applications.

S1R can exist as monomers, dimers, and higher-order oligomers in living cells.^20^ Interestingly, relative oligomer populations are dynamic and can be modulated by S1R ligands.^21^ Therefore, a model of S1R oligomerization and its relationship with receptor function have been postulated.^20,22^ S1R agonists promote the dissociation of S1R into monomers, which can redistribute to other subcellular compartments and chaperone client proteins (i.e., GPCRs, ion channels, transporters), thus modulating the corresponding signaling pathways. In contrast, binding to S1R antagonists prevents these interactions by stabilizing receptor oligomerization, thus preventing the ligand-operated chaperone activity of S1R. However, the available data suggest that the binding of S1R to client partners can vary depending on the biological context in which the S1R ligand (agonist or antagonist) is used. For example, while S1R antagonists promote the dissociation of S1R from NMDA receptors or TRPV1 channels, they improve its binding to μ-opioid receptors.^23,24^ On the contrary, S1R agonists have the opposite effect, promoting the association of S1R with NMDA receptors and other partners.^23^ Nevertheless, the most accepted model suggests that the S1R monomer is the “active” form of the receptor, which is involved in chaperoning client proteins. Accordingly, in the MAM of the ER, S1R exists in a resting inactive state in complex with BiP^25^ and S1R agonists promote the dissociation from BiP, thus favoring the chaperoning activity of the receptor.^25,26^ Our S1R-BiP heteromerization assay revealed that S1R agonist-induced dissociation of S1R-BiP heterodimers was a transient phenomenon. Dissociation reached a peak after 10 min of agonist treatment that decreased to normal levels after 1 h and remained consistent even after 16 h of incubation. Importantly, PRE-084-induced transient dissociation of S1R-BiP was blocked by haloperidol, thus providing pharmacological evidence of the intrinsic activity observed for PRE-084 in the S1R-BiP heteromerization assay. In fact, the transient effect on S1R-BiP heterodimerization may be indicative of a temporary and reversible response of cells to S1R agonists. Therefore, the duration and magnitude of this transient effect will ultimately outline the final physiological response. It should be noted that the effect of PRE-084 on the dissociation of S1R-BiP heterodimers was enhanced by calcium depletion, leading to a reduction in basal heterodimerization even in the presence of haloperidol. These findings provide further validation of the calcium-dependent nature of the S1R-BiP interaction, as previously suggested,^8,25,26^ and agree with the established synergistic effect of S1R agonists and Ca^2+^ depletion that facilitates the association of S1R with IP3R in ER.^8^ On the contrary, haloperidol did not affect the S1R-BiP heteromer content under acute conditions, but prolonged exposure to the antagonist prompted the heterodimerization of S1R-BiP, which agrees with the general hypothesis that S1R antagonists stabilize receptor oligomers. The mechanism by which prolonged treatment with S1R antagonists promotes stabilization of S1R-BiP heterodimers is still unclear. It is possible that these antagonists, either functioning as inverse agonists or not, block the effects of endogenous agonists such as *N*,*N*-dimethyltryptamine^27^ or choline,^28^ thus reducing the chaperoning forms of the receptor (i.e., S1R monomers) and increasing the reservoir of S1R oligomers. However, more research is needed to fully elucidate this mechanism.

Overall, our study describes a simple method capable of predicting the intrinsic activity of S1R ligands. The finding that prolonged incubation with S1R antagonists promotes the formation of S1R-BiP heteromers raises the possibility that this assay could be valuable to guide the development of S1R-targeting drugs with different potency, thus optimizing the intended therapeutic effects of the drug over time.

## Methods

### Drugs

S1R ligands haloperidol (Halo), PRE-084 (PRE), pentazocine (PTZ), and BD-1047 (BD) were purchased from Sigma-Aldrich (St. Louis, MO, USA), and NE-100 (NE), PD-144418 (PD), and 4-IBP were from Tocris Bioscience (Bristol, U.K.). A 10 mM stock solution of each ligand was prepared in DMSO and stored at −20 °C.

### Generation of S1R and BIP NanoBiT-Based Constructs

The pIREShyg3 vector and the pIRESneo3 vector (Clontech Laboratories, Inc.) were modified to contain the cDNA encoding human S1R and/or BiP. Additionally, the cDNA encoding the long or small split halves of nanoluciferase (LgBiT and SmBiT, respectively) was subcloned into the multiple cloning site (MCS) of the original vector pIREShyg3 and pIRESneo3 through unique restriction enzymes *Alf*II and *BstxI*. Furthermore, the mGlu_5_ receptor signal peptide (PS) and the hemagglutinin (HA) epitope cDNA sequences were also included in frame into the 5′ of the MCS to allow plasma membrane trafficking and cell surface detection, respectively. Subsequently, the cDNA encoding the human S1R was amplified by polymerase chain reaction using the primers for S1R (F*Bam*HI 5′-CTAAGA**GGATCC**CAGTGGGCCGTGGGCCGG-3′, R*Eco*RV 5′-ACAGCG**GATATC**AGGGTCCTGGCCAAAGAGG-3′). Amplified human S1R cDNA was then cloned into the *Bam*HI/*Eco*RV sites of pIREShyg3-HA-PS-LgBiT plasmid, thus providing the construct pIRES-S1RLgBiT. Similarly, human BiP was amplified using the primers F*Afl*II 5′-GTCGGC**CTTAAG**ATGAAGCTCTCCCTGGTGGCCGCG-3′ and R*Bam*HI 5′-GTCGGC**GGATCC**CTCATCTTTTTCTGCTGTATCC-3′), and then cloned into the *Afl*II/*Bam*HI sites of pIRESneo3-HA-PS-SmBiT plasmid, thus providing the pIRES-BiP^SmBiT^ construct. All constructs were verified by DNA sequencing.

### Cell Culture and Stable Cell Line Generation

HEK-293 cells were grown in complete cell culture medium consisting of Dulbecco’s modified Eagle’s medium (Sigma-Aldrich, St. Louis, MO, USA) supplemented with 1 mM sodium pyruvate, 2 mM l-glutamine, 100 U/mL penicillin/streptomycin, and 5% (v/v) fetal bovine serum at 37 °C under an atmosphere of 5% CO_2_. HEK293 cells growing in 20 cm^2^ dishes were transiently transfected with DNA encoding for S1R and BiP using polyethylenimine (PEI, 1 mg/mL), as previously described.^29^

A CRISPR-Cas9 S1R HEK-293 knockout cell line (i.e., HEK-293^S1R-KO^ cells) was generated using the σ_1_ receptor (SIGMAR1) human gene knockout kit from Origene (KN201206). Cells were co-transfected with the pCas-Guide vector containing the kit gRNA1 and the donor template vector containing the right and left homologous arms and a puromycin cassette. Cells were selected with puromycin resistance, and knockout clones were identified by immunoblot. Subsequently, a stable HEK-293S1R-KO-S1RLgBiT-BIPSmBiT cell line was generated. To do this, we first performed a dose– response curve for the selection of hygromycin and Geneticin antibiotics in HEK293^S1R-KO^ cells to confirm cell sensitivity to these antibiotics. Therefore, final concentrations of 100 μg/mL and 1 mg/mL were selected for hygromycin and neomycin, respectively. First, the pIREShyg3-HA-PS-S1R^LgBiT^ plasmid was transfected and 24 h after cells were treated with hygromycin (100 μg/mL) for 20–30 days before individual cell clones were selected and secured. Next, a stable cell HEK-293^S1R-KO^ line permanently expressing the S1R^LgBiT^ was transfected with the pIRESneo3-HA-PS-BiP^SmBiT^ plasmid and selected with Geneticin (1 mg/mL, Santa Cruz Biotechnology, Dallas, USA) to generate the doubly S1R^LgBiT^-S1R^SmBiT^ stable cell line. The presence of S1R^LgBiT^ and BIP^SmBiT^ was confirmed using both luminescent measurements and immunoblot analysis.

### Gel Electrophoresis and Immunoblotting

HEK-293 cells were washed in PBS and homogenized in ice-cold 10 mM Tris HCl, pH 7.4 buffer containing a protease inhibitor cocktail (Roche Molecular Systems, Belmont, CA, USA) using a Polytron for three periods of 10 s each. The homogenate was centrifuged at 1000*g* for 10 min at 4 °C. The resulting supernatant was centrifuged at 12 000*g* for 30 min at 4 °C. The membranes were dispersed in 50 mM Tris HCl (pH 7.4) containing a protease inhibitor cocktail. The protein concentration was determined using the BCA protein assay kit (Thermo Fisher Scientific, Inc., Rockford, IL, USA), and 10 μg of protein was used for immunoblotting. Sodium dodecyl sulfate–polyacrylamide gel electrophoresis (SDS/PAGE) was performed using 10% polyacrylamide gels. Proteins were transferred to Hybond-LFP polyvinylidene difluoride (PVDF) membranes (GE Healthcare Europe, Barcelona, Spain) using a Trans-Blot SD semidry transfer cell (Bio-Rad, Hercules, CA, USA). PVDF membranes were blocked with 5% (wt/vol) dry nonfat milk in PBS containing 0.05% Tween-20 (PBS-T) for 45 min and immunoblotted using mouse anti-S1R (1 μg/mL, B-5, sc-137075, Santa Cruz Biotechnology) and mouse anti-BiP/GRP78 (1 μg/mL; BD Biosciences, Franklin Lakes, NJ, USA) antibodies in blocking solution overnight at 4 °C. The PVDF membranes were washed with PBS-T three times (5 min each) before incubation with horseradish peroxidase (HRP) conjugated goat anti-mouse IgG (1/20 000; Pierce Biotechnology, Rockford, IL, USA) in blocking solution at 20 °C for 2 h. After washing the PVDF membranes with PBS-T three times (5 min each) the immunoreactive bands were developed using a chemiluminescent detection kit (Thermo Fisher Scientific) and detected with an Amersham Imager 600 (GE Healthcare).

### S1R-BiP Association Assay

The association between S1R and BiP has been used in the past to identify the functional profile of S1R ligands through a commercial coimmunoprecipitation-coupled enzyme-linked immunosorbent assay (ELISA).^18^ In summary, CHO cells that grow in MEM/Alpha culture medium supplemented with 2 mM Glutamax and 10% (v/v) FBS were treated with the indicated S1R ligands for 30 min at 37 °C. Subsequently, cells were treated with cross-linker dithiobis(succinimidyl propionate) (50 μg/mL) before solubilization and coimmunoprecipitation using rabbit anti-S1R (Abcam, Cambridge, U.K.). The coimmunoprecipitates were analyzed by ELISA as described by the manufacturer.

### NanoBiT Assay

HEK-293^S1R-KO^-S1R^LgBiT^-BIP^SmBiT^ cells were transferred to a white 96-well plate (Corning 96-well, cell culture-treated, flat-bottom microplate) at a density of 90 000 cells/cm^2^. The drugs were added at the indicated concentration before (16 h treatment) or after (2 h treatment) 10 μL of a 10 μM coelenterazine 400a solution (NanoLight Technologies, Pinetop, AZ, USA) was added to each well. After 1 min of incubation either end point (16 h treatment) or time course (2 h treatment) luminescence was recorded using a CLARIOstar Optima plate reader (BMG Labtech GmbH, Ortenberg, Germany) and the output luminescence reported as integrated relative luminescence units (RLU).

### Statistics

Data are represented as the mean ± standard error of mean (SEM). The number of replicas (*n*) and experiments for the condition is indicated in the corresponding figure legend. Outliers were assessed by the Grubbs test. No outliers were found. Comparisons between experimental groups were made using Student’s unpaired *t* test or analysis of variance (ANOVA), followed by Dunnett’s, Tukey’s, or Šídák’s post hoc multiple comparison test using GraphPad Prism 9, as indicated. Statistical difference was accepted when *p* < 0.05.
